# Assessment of availability and challenges of WHO recommended priority life-saving medicines for under five-year children in primary public health facilities of Amhara region

**DOI:** 10.1186/s12887-023-04216-6

**Published:** 2023-08-09

**Authors:** Mastewal Ezezew, Adane Yehualaw, Desalegn Getnet Demsie

**Affiliations:** 1 Ethiopia Pharmaceutical Supply Agency, Bahir Dar, Ethiopia; 2 College of Medicine and Health Sciences, Department of Pharmacy, Bahir Dar University, Bahir Dar, Ethiopia

**Keywords:** WHO priority medicine, Availability, Under five children, Primary public health facility

## Abstract

**Background:**

The world health organization (WHO) priority lifesaving medicines are medicines recommended for the prevention and treatment of leading causes of under-five morbidity and mortality. They should be available in all health systems and at all times. However, the availability of these medicines and its determinants is not well studied in Ethiopia in general and in primary public health facilities Amhara region in particular.

**Objective:**

The study aimed to assess the availability and challenges of the WHO-recommended priority lifesaving medicines for under-five children in primary public health facilities of the Amhara region.

**Methods:**

A cross-sectional study design was conducted from February to December 2020 in 98 health centers and 22 primary hospitals in the Amhara region, Ethiopia. Facilities were selected with a simple random sampling technique. The data were collected through a pretested and structured questionnaire. Binary logistic regression was used to identify predictors associated with availability of WHO-recommended priority lifesaving medicines for under-five children.

**Results:**

The availability of oral rehydration salt was high (82.5%) and the availability of vitamin A (47.5%), morphine tablet (13.3%), and artesunate rectal suppository (7.5%) were within low and very low WHO range respectively. Budget adequacy (AOR = 12.9 CI= (2.1–78.2)), periodic review of stock level ((AOR = 13.4,CI=(1.9–92.0)), training on integrated pharmaceutical logistic system ((AOR = 4.5,CI=(1.0-20.5)), inclusion of WHO priority under five children facility specific medicine list (AOR = 12.4,CI=(2.3–66.4)), lead time for EPSA(Ethiopia Pharmaceutical Supply Agency) procurement (AOR = 7.9,CI=(1.3–44.8)) were significantly associated with availability of all WHO priority lifesaving medicines for under- five children.

**Conclusion:**

The average availability of WHO-recommended priority lifesaving medicines for under-five children was low. The habit of updating bincard and adoption of the life-saving medicine list were the independent predictors of medication availability.

## Introduction

The under-five child morbidity and mortality rate are critical indicators of health improvement, socioeconomic status, and quality of life of a given population and there has been a global drive to improve this -[1–4]. Despite the global progress in reducing child mortality, an estimated 5.2 million children under the age of five died in 2019, of which more than half of those deaths occurred in sub-Saharan Africa [5, 6]. Ethiopia has one of the highest under-five mortality rates with more than 321,000 children under the age of five dying every year [7]. More than 70% of these child deaths are due to five diseases namely pneumonia, diarrhea, malaria, measles and malnutrition which are highly preventable and treatable if lifesaving medicines are available and consistently utilized [7, 8].

With an aim of supporting countries to decrease maternal, newborn and child morbidity and mortality, WHO Department of Essential Medicines and Health Products and other stakeholders developed a list of priority life-saving medicines for women and children [2, 8–13]. Medicines for the management of pneumonia, diarrhea, malaria, neonatal sepsis, HIV, vitamin A deficiency, tuberculosis and pediatric palliative care have been included under priority life-saving medicines for children’s health [8].

This list of medicines is updated every two years with consideration of the global burden of disease, the evidence of efficacy and safety for preventing or treating major causes of newborn and child mortality and morbidity, and comparative cost effectiveness [8, 12]. The medicines listed as priority life-saving medicines are included in the WHO model list of essential medicines (EML), the WHO EML for Children (with the exception of dexamethasone and betamethasone) and WHO treatment guidelines [13–15].

WHO model list of essential medicines provides a guidance for the development of lists of national and institutional essential medicines and therefore countries could be able to select medicines for their own situation. It has also led to the global acceptance of essential medicines as a powerful means of promoting health equity, pursuing universal health coverage (UHC) and ultimately strengthening health systems [12]. In addition to having list of selected priority medicines, WHO also states that essential medicines should be available within the functioning health systems at all times in adequate amounts, in the appropriate dosage forms, with assured quality [8, 12]. Availability of WHO priority life-saving medicine is a measure of the extent to which health facilities have adequate supply of those medicines [16, 17]. WHO promotes the development and mandated use of a national essential medicines list; investments in improving national medicines procurement and supply systems; early screening and accurate diagnosis; the implementation of evidence-based treatment guidelines; implementation of patient-centered treatment initiatives; monitoring medicines using evidence-informed policy-making; and ensuring government commitment and stakeholder engagement in the appropriate use of medicines for improving availability of medicines [12].

In Ethiopia, development of an essential medicine list, promotion of an integrated pharmaceutical logistic system and supply chain management, a drug and therapeutic committee, and guidelines for integrated management of common under-five morbidity are aimed at maximizing availability of WHO life-saving commodities [17–19]. Ensuring availability of quality assured, safe, effective, and affordable essential medicines with which the sector intends to respond for the majority of health problems, and improving rational drug use were among strategic objectives of the health sector transformation plan of Ethiopia [20].

Despite the efforts and promoted actions of WHO, the availability of most of the essential medicines, particularly in the public sector, is still low [12, 21]. In Ethiopia, WHO priority lifesaving medicines for under five children are available in less than 60% of public health facilities. Barriers to the availability of the commodities might be due to stock shortages at EPSA (Ethiopia Pharmaceuticals Supply Agency), unavailability of the products in the market, the supply of products from EPSA with short expiry, insufficient budget for medicines, and absence of training on logistic management [2, 22, 23].

The lifesaving commodities are out of reach for those individuals who need them most despite the strong evidence that their availability contributes a lot in reducing the newborn and child morbidity and mortality [21, 23]. Low availability of WHO lifesaving medicines leads to exacerbation of health system inequities, endangers health, wastes resources and threatens the sustainability of health systems [24]. Evidence showed that maintaining the optimal stock level and avoiding wastages are major challenges in availing life-saving medicines for children [3, 15, 25]. A Study in Uganda reported high stock outs and wastage of priority life-saving medicines due to delays in procurement, poor quantification, late orders from facilities and poor records keeping [26]. Another study in Senegal showed that Stock-out can be linked to the international partners that intervene without coordination in the distribution channels, to the lack of local production of medicines and to insufficient funding [27].

Study reports in Ethiopia as well indicated lower availability and lower strength of life-saving medicines supply chain. For instance, a study in Tigray, Ethiopia has identified that low availability of essential medicines was the main reason hindering access especially in the countryside and the higher prices as compared to IRPs (international reference prices) [3]. In addition, gaps in maintaining stock status were identified in districts like, Jimma, Wollega, Addis Abeba and Gonder [4, 21–23].

Evidence on the availability of Priority Life-Savingmedicines helps managers and policy makers to develop national policy, regulations and strategies to enhance access to them. Few studies have been conducted on a limited number of health facilities on availabilities of WHO priority life-saving medicines for under five children’s in Ethiopia. The study was aimed to assess the availability and challenges of WHO priority life-saving medicines for under five children in primary public health facilities of Amhara region.

## Methods

### Study design, setting and period

Institutional based cross-sectional study was conducted on primary public health facilities of Amhara region from February to December 2020. The Amhara region is one 10 regional states of Ethiopia. The region has a total population of 22,536,999 and has been organized with 15 zones. It currently consists of 4293 public health institutions (8 referral hospitals, 12 general hospitals, 61 primary hospitals, 868 health centers and 3342 health posts), which provide service for the community [28]. Ethiopian Pharmaceutical Supply Agency (EPSA) Bahir Dar, Gondar, Dessie, Addis Ababa No.2 and Adama hubs are the primary source of medicines for those facilities. Addis Ababa is the primary source of medicines, which is located at 552 km from Bahir Dar 253.95 km from Dessie and 78.46 km from Adama.

### Source population and study population

Source population was all public health facilities in Amhara region, while the study population was primary public health facilities of the region. All primary public health facilities which provide service were included in the study. Since ART (Antiretroviral Therapy) drugs were in the list of WHO lifesaving medicines, facilities which do not provide ART service were excluded.

### Sample size determination and sampling procedures

To determine the number of facilities, the public health Logistics Indicators Assessment Tool (LIAT) was used. It recommends taking at least 15% of the total facilities [29]. The sample size for the current study was calculated from the total number of primary public health facilities by taking the inclusion (public health facilities), and exclusion criteria (private health facilities) into account. Accordingly, the total number of primary public health facilities which provide ART service in Amhara region gives 300. Thus, based on the LIAT guideline, the sample size became 120 (i.e., 40% of 300 = 120) [29]. Health facilities were selected by simple random sampling, whereby, all facilities (sampling frame) were assigned numbers from 1 to the last number of facilities on a piece of paper, then the study facilities were selected by lottery method without replacement while discarding those that were outside.

### Selection of medicines

WHO-priority life saving medicines were selected from the essential drug list of Ethiopia. Then availability was assessed by checking the stock status of health commodities from the bincard.

### Data collection materials and procedures

The data was collected using pretested, structured interview and observation checklist. Tools were adapted from Public Health Logistics Indicator Assessment Tool (LIAT), Public Health Logistics System Assessment Tool (LSAT), and indicators developed by John Snow Inc. for United Nation Commission on Life Saving Commodities (UNCoLSC) and various literatures [23, 29, 30]. The data was collected from the head of the health facility, the pharmacy head and the store manager of the health facility. Structured interviews were used to collect the data on health system, health facility and capacity related factors. The observation checklist was used to collect the data on availability of the life-saving MCH (Maternal and Child Health) commodities. All data collection at health facilities was performed by eight trained data collectors. Physical verification was conducted to determine availability on the day of the visit and percentage availability were computed as the proportion of facilities with the WHO recommended priority lifesaving medicines. The data collection process was strictly supervised by the principal investigator.

### Data quality

To assure the quality of data, four-hour training was given to the data collectors regarding the data collection procedures and ethical issues. The questionnaires were also pretested at six health facilities before data collection commenced. Before the data entry, all questionnaires were checked for any missed information. Reliability test was also conducted with all variables and Cronbach’s Alpha value was 0.763.

### Data management and analysis

EPI info was used to enter data, and we used Statistical Package for the Social Sciences (SPSS) version 23 to analyze the data. Descriptive statistical analyses were used to determine frequency counts, averages, and percentages. The results were presented using tables, and texts according to nature of data. To define statistical associations between the dependent and independent variables, binary logistic regression (bivariate and multivariate regression) was used. The cut-off for using predictor variables in the multivariate analysis as pert the recommendation by guideline. Variables in the univariate analysis of p-value < 0.25 was transferred to multivariate analysis. In determining the adjusted odds ratio, a P value less than 0.05 was used to determine statistical significance. WHO’s availability index was used to categorize the availability of medicines from very low to high range. Availability was categorized with a range as.

(i) High means > 80%.
(ii) Fairly high means 50–80%.
(iii) Low means 30–49%.
(iv) Very low means < 30%. However, for the purpose of Binary logistic regression, the average availability of all medicines were categorized as high availability (> 80%) and low availability (≤ 80%), [22, 31].

### Operational definition

- Availability- Medicine was said to be available if it is available in the health facility on the day of data collection.
- Average availability – The mean availability value of eighteen WHO priority medicines for under-five children in each primary public health facilities. The average availability of medicines was categorized as high availability (> 80%) and low availability (≤ 80%).
- WHO recommended priority life-saving Medicines – are 18 medicines selected by WHO as lifesaving for under five children as listed in Table 1.

**Table 1:** Life-saving medicines and their strenght

Drug	Dose
Amoxicillin dispersible tablets	250 mg
Ampicillin injection	250 mg, 500 mg, 1gm
Gentamycin injection	40 mg/mL, 20 mg/mL
Ceftriaxone injection	250 mg, 500 mg, 1gm
Artemether/lumefantrine tablet	120/20 mg
Artesunate injection	50–200 mg
Rectal artesunate	50–200 mg
Oral rehydration salt	Sachet
zinc dispersible tablet	20 mg
Paracetamol oral	100 mg tablet, 125 mg/5mL suspension
Morphine	20–200 mg capsule, 10 mg/mL injection, 10 mg/5 mL oral liquid
Vitamin A	100000IU,200000IU
AZT/3TC tablet	120/60 mg, 60/30 mg
Lpv/r	100/25 mg tablet, 40/10 mg pellet
EFV tablet	50 mg, 200 mg

### Ethical clearance

The study was approved by institutional review board of the College of Medicine and Health Sciences, Bahir Dar University and ethical clearance was provided with a unique identifier of pubh02/12/06/2012 E.C. Permission to conduct the study was also obtained from the officials in-charge of the health facilities. The data collectors oriented during the training. The participants were included based on voluntarism and agreement. Interviews were carried out only with full consent. Before each interview, a clear explanation was given about the aim of the study, and that the information provided by participants was confidential and used only for the purpose of research.

## Results

### Capacity and organizational support

A total of 98 health centers and 22 primary hospitals were involved in the study with a response rate of 100%. Among 120 Health facilities, 64 (53.3%) had IPLS trained professionals. Supportive supervision was provided in 87 (72.5%) of health facilities. Regarding budget availability for medicines, the majority of health facilities 72 (60%) had adequate budget Table 2.

**Table 2:** Capacity and organizational support and health system related characteristics in primary public health facilities of Amhara region, December 2020, (N = 120)

Variable	Category	Frequency (%)	
IPLS training			
	Yes	64 (53.3)	
Supportive supervision of overall Logistics system			
	Yes	87 (72.5)	
Supportive supervision which include child health lifesaving Medicines			
	Yes	46(38.3)	
Budget allocated for Medicines			
	Yes	72(60)	
Availability of national essential medicine list			
	Yes	65(54.2)	
Lead time for EPSA order			
	Less than one month	79 (65.8)	
Lead time for private order			
	Less than one month	87 (72.5)	

*IPLS = Integrated Pharmaceutical Logistics System, EPSA = Ethiopian Pharmaceutical Supply Agency*

With respect to number of staff and their involvement, a mean of number of druggist and pharmacist were 3.45 and 1.13 with standard deviation of 1.14 and 1.1 respectively as elucidated in Table 3.

**Table 3:** Number of pharmacy professionals and their involvement in logistics management system in primary public health facilities of Amhara region, December 2020, (N = 120)

Type of Pharmacy professionals	Mean ± Standard deviation	
Druggist	3.45 ± 1.14	
Pharmacist	1.13 ± 1.1	
Number of all professionals involved in logistic management system		
	3.82 ± 1.9	

### Health system characteristic of health facilities

Our study indicated that 55 (45.8%) of health facilities did not use the national essential medicine list. Most (65.8%) health facilities had a lead time for EPSA procurement of less than one month. Similarly, the lead time for medication from private sources was less than one month for 87 (72.5%) of health facilities (Table 4).

**Table 4:** Selection of medicines in primary public health facilities of Amhara region, December, 2020, (N = 120)

Variable	Category	Frequency (%)
Availability of facility specific medicine list		
	Yes	63 (52.5)
Inclusion of all WHO priority life saving medicines within facility specific medicine list		
	Yes	47 (39.2)
Availability of documented guideline for medicine selection		
	Yes	53(44.2)

*WHO = World Health Organization*

### Selection of medicines

Among a total of 120 selected Primary Health facilities, over half 63 (52.5%) of them had a facility-specific medicine list. However, a majority 73 (60.8%) of health facilities didn’t incorporate the WHO priority medicines for under five children within their facility-specific medicine list (Table 5).

**Table 5:** Forecasting of medicines in primary public health facilities of Amhara region, December, 2020 (N = 120)

Variable	Category	Frequency (%)	
Forecast developed by using dispensed to user data			
	Yes	64 (53.3)	
Forecast developed using issue data			
	Yes	67(55.8)	
Forecast developed using stock on hand			
	Yes	65 (54.2)	
Forecast developed demographic data /prevalence of disease or morbidity			
	Yes	64(53.3)	
Forecast developed using service statistics			
	Yes	61(50.8)	
Forecast validation			
	Yes	47 (39.2)	
Technical assistance provided during forecasting			
	Yes	31(25.8)	

### Forecasting of medicines

To develop forecast/estimate annual need of medicines, 67 (55.8%) and 65 (54.2%) of health facilities used/considered dispensed to user data and issue data, respectively. 73(60.8%) health facilities didn’t validate their forecast by comparing previous estimated consumption with actual consumption. Technical support on how to develop forecasts had been provided for 31(25.8%) of health facilities (Table 6).

**Table 6:** Procurement of medicines in primary public health facilities of Amhara region, December, 2020 (N = 120)

Variable	Category	Frequency (%)
Pharmacist is a member of the tender committee?		
	Yes	61 (50.8)
Short term procurement plan based on forecasted need		
	Yes	98(81.7)
Procedure for placing emergency order		
	Yes	63(52.5)

### Procurement of medicines

While conducting procurement of medicines, 61 (50.8%) of health facilities didn’t officially assign pharmacy professionals as part of a tender committee. The majority (81.7%) of health facilities had procurement plan of medicines based on their forecasted need. (Table 7).

**Table 7:** Logistic management information system of primary public health facilities of Amhara region, December, 2020(N = 120)

Variable	Category	Frequency (%)
Usage of stock card		
	Yes	61 (50.8)
Usage of bin card		
	Yes	63 (52.5)
Usage of report and requisition form		
	Yes	95 (79.2)
RRF include essential logistics data items		
	Stock on hand only	3 (2.5)
	Include all essential logistic data items	117 (97.5)
Automation of logistic management information system		
	Yes	59 (49.2)
How often LMIS report sent to higher level for IPLS integrated medicines		
	Monthly	2 (1.7)
	Bimonthly	96 (80)
	Quarterly	22 (18.3)
How often LMIS report sent to higher level for routine procurement		
	Monthly	44 (36.7)
	Bimonthly	71 (59.2)
	Quarterly	5 (4.2)

*LMIS = Logistics Management Information System ; IPLS = Integrated Pharmaceutical Logistics System*

### Logistics management information system

Assessment of recording tool for logistics information system revealed that 59 (49.2%) of health facility didn’t use a stock record card while, 63 (52.5%) of health facility used a bin card for recording essential data items of all medicines. Our finding also showed that 117 (97.5%) of health facilities report all essential data items (stock on-hand, quantity issued and loss adjustment) to higher level of facility (Table 8).

**Table 8:** Inventory management practice in primary public health facility of Amhara region, December, 2020 (N = 120)

Variable	Category	Frequency (%)
Maximum and minimum stock level policy established		
	Yes	111 (92.5)
Stock level currently falls between maximum and minimum level		
	Yes	87 (72.5)
Stock level reviewed periodically		
	Yes	41 (34.2)
Written storage guideline available		
	Yes	91 (75.8)
Health facility store and issue with FEFO principle		
	Yes	115 (95.8)
Health facility conducts physical inventory		
	Yes	58 (48.3)
Existing storage capacity adequate to ensure that no products are out of stock		
	Yes	110 (91.7)
Visual inspection of products conducted at storage		
	Yes	89 (74.2)

*FEFO = First Expire First Store*

### Inventory management

Of the 120 primary health facilities, the majority 111 (92.5%) of them established policies and guidelines for maximum and minimum stock level that should be maintained and in 87 (72.5%) the stock level fell between the maximum and minimum range. Among all health facilities 79 (65.8%) of them didn’t review their stock level periodically. 91(75.8%) of health facilities had a written procedure for storing and handling of medicines. The existing storage capacity was adequate to handle quantities needed to ensure that no stock shortages occur in 110 (91.7%) of health facilities (Table 9**)**.

**Table 9:** Availability of WHO-recommended priority lifesaving medicines for under-five children in primary public health facilities of Amhara region November 2020 (N = 120)

WHO leading cause of morbidity and mortality	WHO-recommended priority lifesaving medicines	Aggregate availability N (%)	WHO availability Index
Pneumonia and sepsis management	Amoxicillin 250 mg dispersable Tablet	96 ( 80% )	Fairly high
	Gentamycin injection (40 mg/ml,20 mg/ml)	77 (64.2%)	Fairly high
	Ampicilin injection (250 mg,500 mg ,1gm)	69 (57.5%)	Fairly high
	Ceftriaxone injection(250 mg,500 mg,1gm)	92 (76.7%)	Fairly high
Diarrhea	Oral Rehydration salt (sachet)	99 (82.5% )	high
	Zinc sulphate 20 mg dispersable tablet	89 (74.2%)	Fairly high
Malaria	Arthemather /lumifantrine (20/120 mg)	78 ( 65% )	Fairly high
	Artesunate injection (50–200) mg	67 (55.8%)	Fairly high
	Artesunate rectal (50–200) mg	9(7.5% )	Very low
Pain Management	Paracetamol oral dosage form (100 mg tablet and 120 mg/5 ml suspension )	72 (60%)	Fairly high
	Morphine tablet (20–200) mg capsule, 10 mg/ml injection, 10 mg/5 ml oral liquid	16 (13.3%)	Very low
Vitamin A deficiency	Vitamin A capsule 100,000&200,000 IU	57 (47.5%)	low
HIV/AIDS	AZT/3TC (120/60) mg tablet	89 (74.2%)	Fairly high
	Lop/r (100/25)mg tablet	77(64.2%)	Fairly high
	Lop/r (40/10)mg pellet	91 (75.8%)	Fairly high
	AZT/3TC (60/30) mg tablet	85 (70.8%)	Fairly high
	EFV120 mg tablet	87 (72.5%)	Fairly high
	EFV 50 mg tablet	76 (63.3%)	Fairly high

### Availability of medicines

Oral rehydration salt was available in 99 (82.5%) of health facilities. Similarly, amoxicillin dispersible tablet (20 mg), ceftriaxone injection, and Lop/r (40/10 mg) pellet were available in 96(80%), 92 (76.7%), 91 (75.8%), of health facilities respectively. However, artesunate rectal was available in 9 (7.5%) of health facility and was also not available in any hospital. (Table 10)

### Average availability of WHO priority medicines for under five children

Health facilities had minimum 5 (27%) and maximum 17(94%) life-saving medicines with mean value of 11.050 (61.4%). Then health facilities which had greater than 80% of WHO priority life-saving medicines were categorized as high availability and less than or equals to 80% as low availability [5, 13]. Based on this, 73(60.8%) of health facility had low availability of medicines whereas 47(39.2%) of them had high availability.

### Predictors of availability of WHO priority lifesaving medicines for under-five children

Without controlling for confounders, some variables were significantly associated with availability of WHO priority life-saving medicines for under five children. At a bivariate level of analysis, level of health facility, inclusion of WHO priority under five children medicines with facility specific medicine list, technical assistance during forecast, lead time for EPSA procurement, periodic review of stock level, IPLS training, forecasting validation, and budget adequacy were significantly associated with availability of WHO priority life-saving medicines for under five children (p < 0.25).

In a multivariate logistic model, having other variables controlled, budget adequacy (p = 0.005), inclusion of WHO priority under-five children medicines within the facility-specific medicine list (p = 0.003), lead time for EPSA procurement (p = 0.02), Integrated pharmaceutical logistics system training (p = 0.048), periodic review of stock status (0.008) and level of health facility (p = 0.00) were significantly associated with availability of WHO priority life-saving medicines for under five children.

Health facilities which review their stock status periodically were 13 times more likely to have high availability of WHO priority life-saving medicines for under five children. (AOR = 13.4, CI (1.96–92.04). However, hospitals were 0.009 times less likely to have high availability as compared to Health centers (AOR = 0.009, 95% CI= (0.001–0.122)). (Table 10)

**Table 10:** Predictor of availability of WHO priority Life saving medicines for under five children in primary public health facilities of Amhara region, December 2020 (N = 120)

Variable (N = 120)	Average availability		95% Confidence interval		P value				
	High (%)	Low (%)	COR	AOR					
Level of health facility									
Heath center	44(44.9%)	54(55.1%)	1	1					
Hospital	3 (13.6%)	19(86.4%)	0.19(0.05–0.69)	0.01 (0.001–0.12)	0.00				
Adequacy of budget									
Yes	34(47.2%)	38(52.8%)	2.84(1.28–6.32)	12.98(2.15–78.24)	0.005				
No	13(27.1%)	35(72.9%)	1	1					
Technical Assistance									
Yes	24(77.4%)	7(22.6%)	7.79(3.07–19.75)	7.85 (0.98–63.20)	0.053				
No	23(25.8%)	66(74.2%)	1	1					
Inclusion of WHO priority under five children medicines with facility specific medicine list									
Yes	34(72.3%)	13(27.7%)	12.1(5.03–28.99)	12.48(2.34–66.48)		0.003			
No	13(17.8%)	60(82.2%)	1	1					
Lead time For EPSA procurement									
Below one month	25(31.6%)	54(68.4%)	1	1					
One month and above	22(53.7%)	19(46.3%)	2.5(1.15–5.43)	7.90(1.39–44.89)	0.02				
Periodic review of stock level									
Yes	28(68.3%)	13(31.7%)	6.8(2.95–15.69)	13.43(1.96–92.04)			0.008		
No	19(24.1%)	60(75.9%)	1	1					
IPLS training									
Yes	29(45.3%)	35(54.7%)	2.02(0.95–4.29)	4.57 (1.02–20.55)	0.048				
No	18(32.1%)	38(67.9%)	1	1					
Forecasting validation									
Yes	14(29.8%)	33(70.2%)	0.514(0.24–1.12)	1.167(0.29–4.65)	0.826				
No	33(45.2%)	40(54.8%)	1	1					

*WHO = World Health Organization; IPLS = Integrated Pharmaceutical Logistics System; EPSA = Ethiopian Pharmaceutical Supply Agency;*

## Discussion

WHO advocates medicines which respond to the priority health need of specific population group should be available at all times in adequate amounts, be affordable, and have a proven efficacy, quality and safety [8]. Since 2005, Ethiopia also introduced an integrated pharmaceutical logistic system and makes every effort to integrate child health medicines supply chain management with integrated pharmaceutical logistic system with intention of improving availability of priority life-saving child health medicines [18, 32]. However, in the primary public health facilities of Amhara Region, utilization (prescribing pattern) of those WHO priority medicines for treatment of the top causes of under-five children morbidity and mortality is still low [33]. Here, this study assesses availability of WHO priority medicines for under-five children and associated factors in primary public health facilities of Amhara Region.

### Availability of WHO recommended life-saving medicines for under five children

The average availability of WHO priority medicines were 61.4% which was low availability. This might be due to inability to review stock level periodically and failure to consider WHO priority medicines for under-five children within the facility specific drug list and poor supportive supervision [23, 34]. The low availability of these WHO recommended life-saving priority medicines could be a disaster to child survival. Medications should be available at sufficient quantities in Health facilities to save millions children’s lives [27]. The low availability of medicines at public health facilities could also enforce patients to purchase medicines from private pharmacies where quite often are sold for higher price that lead the patients to dig deep into their pockets to pay for medicines [2]. Due to variations in medicine pricing policy, methodology, types of prevalent diseases and medicine supply systems it is difficult to make a comparative analysis of medicines availability. However, the finding was comparable with the finding of the study done in Jimma zone (south-west Ethiopia),(68%) [21] and higher than findings in Tigray region, Ethiopia (41.9%) [2].

#### Pneumonia and sepsis management

This study revealed that gentamicin injection (40 mg/ml, 20 mg/ml), ampicillin injection (250 mg, 500 mg, and 1 gm) and ceftriaxone injection (250 mg, 500 mg, and 1 gm) were available in 64.1%, 57.5%, and 76.75 of the health facilities, respectively. As pneumonia-related morbidity is an important part of outpatient visits and hospitalizations in low and middle income countries [27] and pneumonia is the foremost causes of death and disease burden among under-five children in Ethiopia [2]. These drugs should be availed sufficiently as they have high impact on child survival.

The respective stock levels were lower than the percentage availability in Jimma (94.4%, gentamicin;77.8% ampicillin;88.9% ceftriaxone) [21], Tigray regions (90%,gentamicin; 60%,ampicillin;100%,ceftriaxone) [2], Ethiopia. Considering WHO availability index range, study finding in Addis Ababa (74.3%) [23], was in similar availability index range with the current study result. However, this study finding was higher than study finding in Wollega zone, western part of Ethiopia in 2016 (0%) [22] and Jinja district of Uganda (37.5%,gentamicin;18.7%,ampicillin;9.4%,ceftriaxone) [35]. The higher deviation of result of the current study and study finding in Wollega zone, might be due to strong supply chain management system in Amahara region following consideration of the drug as life saving [32]. This resulted in routine deliveries of these life-saving medications to health facilities with other program commodities and improved access and visibility of data related to child health [32]. Coordination in the distribution channels of the health system can improve the availability of life-saving medications [27].

#### Diarrhea management

Both WHO and Ethiopian treatment guidelines recommend combination of ORS and zinc as the best treatment options. The current study found that Oral rehydration salt (sachet) was available in 82.5% of health facilities, which was lower than the report in Jimma[21], Addis Ababa [23] and Tigray[2]. However, this result is higher than study finding of Wollega zone, Western Ethiopia in 2016 (40%) [22] .

This study also revealed that, availability of zinc dispersible tablet (20 mg) was 74.2%, which were in the same WHO availability index range [21, 36] with study finding of Addis Ababa city administration (54.3%) [23] However the current finding was lower than the study finding of Guediawaye and Pete Health district of Senegal (84.6%) [27].

#### Malaria management

This study demonstrated that percentage availability of artemether/lumefantrine was 65%. The result was lower than the availability in Wollega (66.7%) [22], but higher than the findings in Jimma (61.1%) [21] and Tigray (60%) [2] regional states. The fining was also higher than reports in Uganda (0%)[20] and Senegal (46%) [27]. Artesunate (50%) was better available in Amhara region than Jimma [21] and Tigray[2]. Low availability and the spread resistance of antimalarial drugs have major consequences for malaria control in tropical Africa, and Ethiopia in particular [37–39].

#### Pain management

Despite WHO recommendation of Morphine preparation (oral and IM preparation) for pain management of under five children [8], it was available only 13.3% of selected health facilities which was higher than finding of study in Tigray region, Ethiopia (0%) [2]. This large deficit in availability might be due to severely restricted formularies of opioids, poor availability of opioids even when included in national essential medicines lists and excessive regulation that limits patient access or could be partly tied to the country’s standard treatment guideline which makes pethidine as the first line for pain management as compared to morphine, which is listed as the alternative treatments for pain management [2]. Due to the ever-increasing burden of cancer and other causes of sever pain, and the rising need of opioids in developing countries, low availability of these drug can be thought as a failure to implement appropriate logistics or supply chain principles, or there is budget constraint to procure drugs [40–42].

Paracetamol oral dosage form (100 mg tablet and 125 mg/5ml suspension) was available in 60% of selected health facilities that is comparable with study finding in Tigray Region ,Ethiopia (80%)[2]. According to this study, the availability of both paracetamol 100 mg tablet and 125 mg/5 ml (60%) were lower than the finding of study in Jimma zone, South West Ethiopia, (94.4%) for only paracetamol tablet preparation [21]. This difference of the current study with study findings of Jimma zone might be due to non-inclusion of paracetamol oral dosage forms (suspension and tablet) with facility specific medicine list in most health facilities.

#### Vitamin A deficiency management

This study also point out that, Vitamin A (100000IU, 200000IU) was available in 47.5% of health facilities which was far lower than study finding in Guediawaye and Pete health districts of Senegal (84.6%) [27]. this availability difference might be accounted by unable to integrate vitamin A supply chain management with Integrated pharmaceutical logistic system at national level [32]. The low availability of vitamin A could lead to missed opportunities for supplementation and detrimental impact on the health of eye of the children. Health professionals’ difficulty in understanding the clinical efficacy of vitamin A and poor maternal demand may be the cause of its low availability [27]. Failure to avail Vitamin A can result in harm, especially pregnant women and children in Ethiopia or other countries with weak supply chain management system [43, 44].

HIV/AIDS Management: Regarding to the current study, AZT/3TC (60/30 mg) and EFV 50 mg were available in 70.8% and 63.3% of health facilities. According to this finding, availability of AZT/3TC (60/30 mg) was lower than and EFV 50 mg was comparable with the study finding in Jimma zone, south west Ethiopia, (88.9%, 72.2%) [21]. Similarly, this study found that, the availability of AZT/3TC (120/60 mg), Lop/r (100/25 mg), Lop/r (40/60 mg) and EFV 120 mg were 74.2%, 64.2%, 75.8% and 72.5% respectively. According to WHO availability index, availability of all those medicines were fairly high.

However similar study on availability of AZT/3TC (120/60 mg), Lop/r (100/25 mg), Lop/r (40/60 mg) and EFV 120 mg were not found. This might be due to; those medicines were considered as pediatrics first line treatment recently [45].

### Predictor of availability of WHO priority Medicines for under-five children

Availability of WHO priority lifesaving medicines was related to a number of factors, including budget, lead time for procurement, IPLS training, periodic review of stocks, and inclusion of medications within the facility specific medicine list. The association was significant as evidenced by p-values of budget (p = 0.005), lead time for EPSA procurement (p = 0.02), getting IPLS training (p = 0.048), periodic review of stock level of medicines (P = 0.008), and inclusion of WHO priority lifesaving medicines for under five children within facility specific medicine list (p = 0.003. This study found that, there was significant association between Availability of WHO priority medicine for under five children and adequacy of budget (p = 0.005). According to this study health facilities which have adequate budget were 12 times more likely to have high availability of WHO priority life-saving medicines for under-five children. This study finding was supported with study done in Addis Ababa where adequacy of budget have association with availability of life-saving maternal and child health commodities (p = 0.003) [23]. Study finding in Uganda and Kenya [26, 46, 47] also found that funds significantly predict availability of essential medicines, which is in agreement to the current study finding. Systemic review which was conducted in Indonesia also showed that budget constraint as a factor for low availabilities of essential medicines in public health sectors [28]. Similarly, UN Commission on Life-Saving Commodities for Women and Children also identify budget constraint particularly in key commodities and supply chain system as factor affecting availability[48].

This study also revealed that lead time for EPSA procurement (p = 0.02) was significantly associated with availability of WHO priority medicines for under-five children. This finding was in line with finding of study conducted in Uganda [26] which revealed that lead time significantly associate with availability of essential medicines in public health facilities of Uganda.

Getting IPLS training were also significantly associated with availability of WHO priority life-saving medicines for under-five children (p = 0.048). Based on the finding health facilities which have IPLS trained pharmacy professionals were 4.57 times more likely to have high availability. Integrated pharmaceutical logistic system (IPLS) aims to ensure that patients always get medicines they need through improving sustainable availability of quality assured medicines. Training on IPLS promotes implementation of IPLS to achieve the ultimate purpose of improving availability of priority life-saving medicines via improving supply chain data quality [12, 40].

This study also revealed that periodic review of stock level of medicines was associated with availability of WHO priority life-saving medicines for under five children (P = 0.008). Health facilities that review their stock status periodically were 13 times more likely to have high availability of those medicines than those which didn’t review their stock status periodically. The best way to ensure no stock out of medicines at health facility is periodic review of stock level. This is because ,periodic review of stock level informs professionals in charge of managing medicines supply chain system ,when to order or issue, how much to order or issue, and how to maintain an appropriate stock level of all medicines to avoid shortages and oversupply [49].

This study also found that inclusion of WHO priority life-saving medicines for under five children with facility specific medicine list were significantly associated with Availability of WHO priority medicine for under five children (p = 0.003). Health facilities which include WHO priority life-saving medicines for under-five into their facility specific medicine list were 12 times more likely to have high availability of medicines as compared to those facilities which didn’t include into their facility specific medicine list. This is might be due to, having facility specific medicine list promotes consistent and prioritized procurement for only medicines which was considered within the list ,which in turn improve availability of considered medicines [17].

Regarding level of health facility, our finding showed that as compared to health centers, hospitals were 0.009 times less likely to have high availability of WHO priority medicines for under-five children. This finding was in contrast with study finding in Uganda [27] that revealed, availability of higher level facilities were better as compared to lower level of facilities. This deviation might be due the fact that, in Ethiopia ministry of health in collaboration with UNICEF and other donors allocate life-saving medicines for health centers every year through woreda health office.

Even though there is a reduction in child mortality, the findings from this study advocate that availability of priority life-saving medicines for children is still low. The low availability of life-saving medications may be related to budget insufficiency, failure to estimate lead time for procurement, lack of IPLS training and failure to periodic review of stocks. Therefore, country or regional health authorities must therefore improve the availability of priority life-saving medicines in the public sector by monitoring efficiency of the public sector procurement system, allocating sufficient budget as well as encouraging local pharmaceutical manufacturing. In general, this study suggests the regional priority life-saving medicine policy to be established, developed and enforced at public health facilities to ensure availability to basic health services.

### Limitation of the study

This study measured the point availability of life-saving medicines. This may affect generalizability of the findings. The study facilities were only from a public health facility perspective and did not include other stakeholders such as Ethiopian pharmaceutical supply agency, Regional Health bureau, Zonal health department, and Woreda Health office and private health facilities.

### Conclusion and recommendation

In general, the average availability of WHO-recommended priority lifesaving medicines for children under five was low on the day of the visit. Most medicines were within the high and fairly high WHO availability index ranges; however, vitamin A capsule were “low” while morphine tablet and artesunate rectal suppository were rated as “very low”. The habit of updating bincard and adoption of the life-saving medicine list were the independent predictors of medication availability. EPSA should train the staffs, inform woreda health office and regional health bureau the need for robust supply chain system. In addition, EPSA should work with international suppliers and charity organizations to get funds for medicines procurement.

### Ethics approval

The study was approved by institutional review board of, collage of medicine and health sciences and was conducted according to the Declaration of Helsinki and Good Clinical Practice. https://www.wma.net/policies-post/wma-declaration-of-helsinki-ethical-principles-for-medical-research-involving-human-subjects/. Informed consent was obtained from all subjects and/or their legal guardian(s).

## Data Availability

The datasets generated and/or analyzed during the current study are available and will be provided by the corresponding author whenever requested.
